# Rhizosphere Reactive Oxygen Species: Detection Methods, Distribution Characteristics, Production Mechanisms, and Environmental Effects

**DOI:** 10.3390/cimb48080758

**Published:** 2026-07-26

**Authors:** Xiaoling Xu, Chuanxiang Li, Jian He, Jian Wang, Jinbo Liu

**Affiliations:** School of Petroleum Engineering and Environmental Engineering, Yan’an University, Yan’an 716000, China; xuxiaoling0309@163.com (X.X.); 15634129251@163.com (C.L.); hej19@lzu.edu.cn (J.H.); wangjian@yau.edu.cn (J.W.)

**Keywords:** rhizosphere, reactive oxygen species, detection methods, characteristics, environmental effects

## Abstract

The rhizosphere, the zone around the roots of plants, including the root, the soil on the root itself, and the surrounding area soil, is the hotspot for the production of reactive oxygen species (ROS). Functioning as redox signaling mediators, rhizosphere ROS regulate a series of key processes in the rhizosphere microenvironment. These biological and geochemical events cover root morphogenesis, the formation of beneficial plant–microbe symbioses, and element biogeochemical transformations. Nevertheless, the pronounced spatial heterogeneity, drastic redox fluctuations, and intricate interfacial interactions within the rhizosphere impede accurate in situ detection of ROS. Beyond technical barriers, the coupled regulatory effects exerted by biotic and abiotic factors on ROS dynamics, together with the associated ecological outcomes induced by rhizosphere ROS, remain difficult to fully disentangle. This review first discusses updated strategies and optimized methodologies for in situ rhizosphere ROS monitoring. We then characterize the spatial distribution patterns and microscale hotspots of rhizosphere ROS. Importantly, this review dissects the complex coupled regulatory network formed by biotic, abiotic, and environmental factors and molecular regulatory pathways that control ROS production. Furthermore, this study systematically illustrates diverse environmental effects triggered by rhizosphere ROS. Finally, the present work identifies prevailing research gaps and unresolved limitations in current studies. On this basis, we further propose targeted research directions for future studies in this field, providing comprehensive theoretical evidence to deepen the understanding of rhizosphere ROS formation and their mediated biogeochemical processes.

## 1. Introduction

The rhizosphere, as the pivotal zone for material exchange, energy flux, and signal transduction among plant roots, soil microorganisms, and the surrounding soil environment, is the most active micro-interface in the soil ecosystem [1]. Different from the bulk soil, the rhizosphere functions as both the principal site of root water and nutrient acquisition and the frontline barrier protecting plants from pathogenic, drought, and heavy metal stresses [2,3]. The rhizosphere harbors diverse microorganisms including bacteria, fungi, and actinomycetes, as well as abundant root exudates, forming a tightly interacting microecosystem [4]. Within this highly dynamic microecosystem, intense redox reactions take place at the interfaces between roots, microbes, and soil particles, generating a large quantity of reactive molecules [5]. Among them, reactive oxygen species (ROS) act as the key active substances that govern the intricate biogeochemical processes occurring within the rhizosphere.

ROS are a series of chemically active oxygen-containing molecules or free radicals produced by the incomplete reduction of molecular oxygen, including superoxide radicals (O_2_^•−^), hydrogen peroxide (H_2_O_2_), and hydroxyl radicals (^•^OH) [6]. ROS production is traditionally regarded as being associated with photochemical processes in surface water, atmospheric particles, as well as the upper soil layer, thus leaving the role of ROS in soil biogeochemical processes overlooked because of limited sunlight penetration depth (<0.5 mm) [7]. Recently, ROS generation under dark conditions has also been explored, which was suggested to be related to the redox reactions at dynamic interfaces such as the rhizosphere [8]. As a representative micro-interface with distinctive biochemical features, the rhizosphere supports robust ROS formation driven by active radial oxygen loss, abundant root exudates, and intensive microbial redox metabolism, making it the dominant hotspot for soil ROS production [9,10]. Generally, rhizosphere ROS formation arises from synergistic modulation by plant roots, soil microorganisms, and soil mineral fractions. Their ecological effects are involved in regulating root growth and development, shaping the rhizosphere microbial community structure, driving the circulation of elements (e.g., carbon) and sulfur, and promoting the transformation and degradation of organic pollutants [11]. These diverse ecological functions highlight the pivotal role of rhizosphere ROS dynamics. However, due to the rhizosphere’s intricate structure and pronounced spatial heterogeneity, it remains challenging to unravel the molecular mechanisms and spatiotemporal dynamics governing rhizosphere ROS. Furthermore, these challenges are further compounded by the inherently low concentrations and short lifetimes of rhizosphere ROS [6]. Therefore, although the importance of rhizosphere ROS has been widely recognized, the research in this field still faces many challenges. Conventional ex situ detection methods cannot maintain the original state of the rhizosphere microenvironment during the sampling and testing process [8]. Therefore, it is impossible to accurately capture the real-time dynamic information of ROS. Meanwhile, rhizosphere ROS generation arises from coupled interactions between diverse biotic and abiotic factors [12,13]. However, previous studies mostly focused on the independent effects of a single factor such as roots, microorganisms, or soil components, lacking an in-depth understanding of the coupled regulation mode and synergistic mechanism. More importantly, existing studies have largely centered on either the toxic effects of ROS or on isolated regulatory functions in plant–microbe–soil interactions, lacking a systematic elaboration of the rhizosphere ROS dynamic balance regulation network and its multi-level ecological effects [12].

This work synthesizes recent advances to establish an integrated research framework for rhizosphere ROS research. Specifically, this review aims to systematically elaborate the in situ detection methods, distribution characteristics, and hotspot distribution of rhizosphere ROS, as well as the coupled regulation mechanism of biotic and abiotic factors and their multi-level environmental effects. Moreover, this study proposes perspectives for future research, hoping to provide theoretical support for an in-depth understanding of the generation process and ecological functions of rhizosphere ROS.

## 2. Methods for Detecting Rhizosphere ROS

The accurate determination of rhizosphere ROS is the prerequisite for clarifying its distribution characteristics, analyzing its formation mechanism, and evaluating its ecological effects. According to the technical principle, the commonly used rhizosphere ROS detection technologies can be generally divided into fluorescence imaging detection technology, chemiluminescence detection technology, and electron paramagnetic resonance spectroscopy detection technology.

### 2.1. Fluorescence Imaging Detection Technology

Fluorescence imaging is a prevailing technique for rhizosphere ROS detection. It relies on ROS-specific fluorescent probes, which react with target ROS to produce fluorescent products [12]. Fluorescence signals are then measured to achieve qualitative and quantitative analysis of rhizosphere ROS [9,13]. Owing to its high sensitivity and specificity, this approach visualizes the spatial distribution and dynamic variations in rhizosphere ROS.

Based on their properties and modes of action, fluorescent probes are categorized as small-molecule or genetically encoded [14]. Among them, small-molecule fluorescent probes are widely used, owing to their low cost, reliable stability, and simple operation [15]. The most used probe is 2′,7′-dichlorodihydrofluorescein diacetate (H_2_DCFDA), which can react with ROS to generate a highly fluorescent dichlorofluorescein (DCF) [16]. In addition, several small-molecule probes have been developed for different ROS. For instance, 4-aminoantipyrine and scopoletin are applied specifically to detect H_2_O_2_ [17,18]. Notably, small-molecule fluorescent probes are commonly paired with in situ imaging tools, such as confocal laser scanning microscopy. For example, researchers have coupled the microfluidic chip technology with a confocal laser scanning microscopy platform, building an integrated in situ real-time fluorescence imaging detection system for the detection of ROS in the rice rhizosphere [19]. Notably, small-molecule fluorescent probes have notable limitations [20]. Their performance is susceptible to rhizosphere pH, temperature, and chemical conditions [21]. To address these drawbacks, genetically encoded fluorescent probes have been developed recently. The representative probe is the roGFP2-Orp1 fusion probe [22]. It can monitor the dynamic changes in H_2_O_2_ in the rhizosphere in a long-term and targeted manner [23,24]. Nevertheless, high technical requirements, complicated preparation, and considerable costs restrict its application, which is currently limited to rhizosphere ROS detection of model plants like Arabidopsis thaliana [25].

### 2.2. Chemiluminescence Detection Technology

As a key method for rhizosphere ROS detection, chemiluminescence relies on reactions between specialized reagents and ROS that produce excited molecules through energy conversion [26]. The photons emitted during molecular de-excitation are detected to quantify target ROS [26,27]. Compared with fluorescence imaging detection technology, chemiluminescence detection technology does not require excitation light sources such as external fluorescence lamps or lasers, which effectively avoids the interference of background fluorescence and scattered light in the rhizosphere environment and the photobleaching effect of fluorescent probes [28,29,30]. It has the advantages of a high signal-to-noise ratio, wide linear range, and low detection limit, and is suitable for the quantitative detection of ROS [31].

The chemiluminescent probes currently used in rhizosphere ROS are mainly derivatives of lucigenin and luminol [32]. Lucigenin has a high reaction specificity with O_2_^•−^ and is often used for the quantitative detection of O_2_^•−^. Luminol, as a commonly used general-purpose chemiluminescent probe, can react with various ROS components such as H_2_O_2_ and ^•^OH to generate a strong optical signal, and it is suitable for the quantitative determination of total rhizosphere ROS [33,34]. However, chemiluminescence detection technology also has obvious technical defects. On the one hand, the signal intensity of chemiluminescence detection is easily affected by environmental factors such as the pH value of the rhizosphere soil, reducing the accuracy and repeatability [35]. On the other hand, the spatial resolution of chemiluminescence detection technology is relatively low. It can only obtain the total ROS content, but cannot accurately distinguish the specific source location and spatial distribution information of ROS [35].

### 2.3. Electron Paramagnetic Resonance Spectroscopy Detection Technology

Electron paramagnetic resonance (EPR) spectroscopy can achieve the direct qualitative and quantitative detection of rhizosphere ROS. EPR/ESR spectroscopy captures free radicals via spin trapping. External magnetic fields split the unpaired electrons of radicals to generate measurable spin transition signals [36]. Variations in resonance peak positions and intensities allow differentiation and quantification of individual free radical components. EPR spectroscopy can not only achieve the direct detection of ROS but also effectively distinguish different ROS through spectral fingerprint information [37]. Notably, EPR spectroscopy also has obvious limitations. On the one hand, the instrument is expensive, large in size, and difficult to operate. It is difficult to achieve real-time detection of rhizosphere ROS under natural field conditions [38]. On the other hand, the half-life time of ROS is extremely short, usually only in the order of microseconds or even nanoseconds. Direct detection is often difficult to achieve in actual research. It is necessary to add a spin-trapping agent to the rhizosphere soil sample in advance. The spin-trapping agent can react with the short-lived free radical components in the rhizosphere to generate a relatively stable spin adduct, which can be stably captured by the EPR spectrometer. The 5-tert-butoxycarbonyl-5-methyl-1-pyrroline N-oxide (DMPO) has a strong trapping effect on ROS and is the most widely used spin-trapping agent [39,40]. However, it should be noted that EPR spectroscopy is primarily adopted to verify rhizosphere ROS and identify ROS types.

### 2.4. High-Performance Liquid Chromatography Detection Technology

High-performance liquid chromatography (HPLC) represents a precise ex situ quantitative method for rhizosphere ROS, widely applied to differentiate and quantify individual radical species after probe trapping reactions [4,15]. The principle relies on trapping specific ROS with chemical reagents to generate characteristic derivatives. Typical trapping systems include terephthalic acid for ^•^OH and hydroethidine for O_2_^•−^. Their oxidation products exhibit distinct retention times, enabling accurate discrimination of single ROS that overlap under single optical detection [5,8]. Compared with single optical assays, HPLC eliminates spectral cross-interference between multiple ROS derivatives and delivers superior quantification accuracy for soil extract samples. This technology possesses prominent strengths including high separation efficiency, low detection limits, and stable quantitative linearity, supporting comparative analysis of ROS pools across different rhizosphere zones [15]. However, HPLC requires destructive soil sampling and pre-extraction, which destroys intact rhizosphere micro-interfaces and loses real-time spatial dynamic information. Meanwhile, instrument costs and complex pre-processing steps restrict large-scale batch field sample analysis.

### 2.5. Integrated In Situ Imaging Technology

Integrated in situ imaging platforms emerge as comprehensive multi-dimensional tools to overcome the single-mode defects of the above independent detection techniques, realizing synchronous visualization and quantification of rhizosphere ROS alongside coupled microenvironmental parameters [10,11]. Representative systems combine microfluidic rhizosphere chips (iRhizo-Chip), confocal fluorescence modules, planar optodes, and microelectrode arrays into one unified detection setup. H_2_DCFDA hydrogel probes embedded inside microfluidic devices capture real-time ROS fluorescence signals, while matching oxygen/pH microelectrodes synchronously record rhizosphere physicochemical gradients that regulate ROS generation [11,12]. Such platforms visualize ROS hotspots at root apices and root–soil interfaces with micrometer spatial resolution, and record diurnal ROS fluctuation dynamics under simulated light and water regimes [19]. The advantages of integrated in situ imaging lie in non-destructive, continuous observation of intact plant–root–soil systems and simultaneous acquisition of ROS distribution and co-occurring environmental factors, which helps disentangle coupled abiotic regulatory effects on rhizosphere ROS [23]. Current limitations center on strict laboratory cultivation conditions, as most platforms cannot be deployed under complex natural field soils and microfluidic chip fabrication costs remain high.

**Figure 1:**
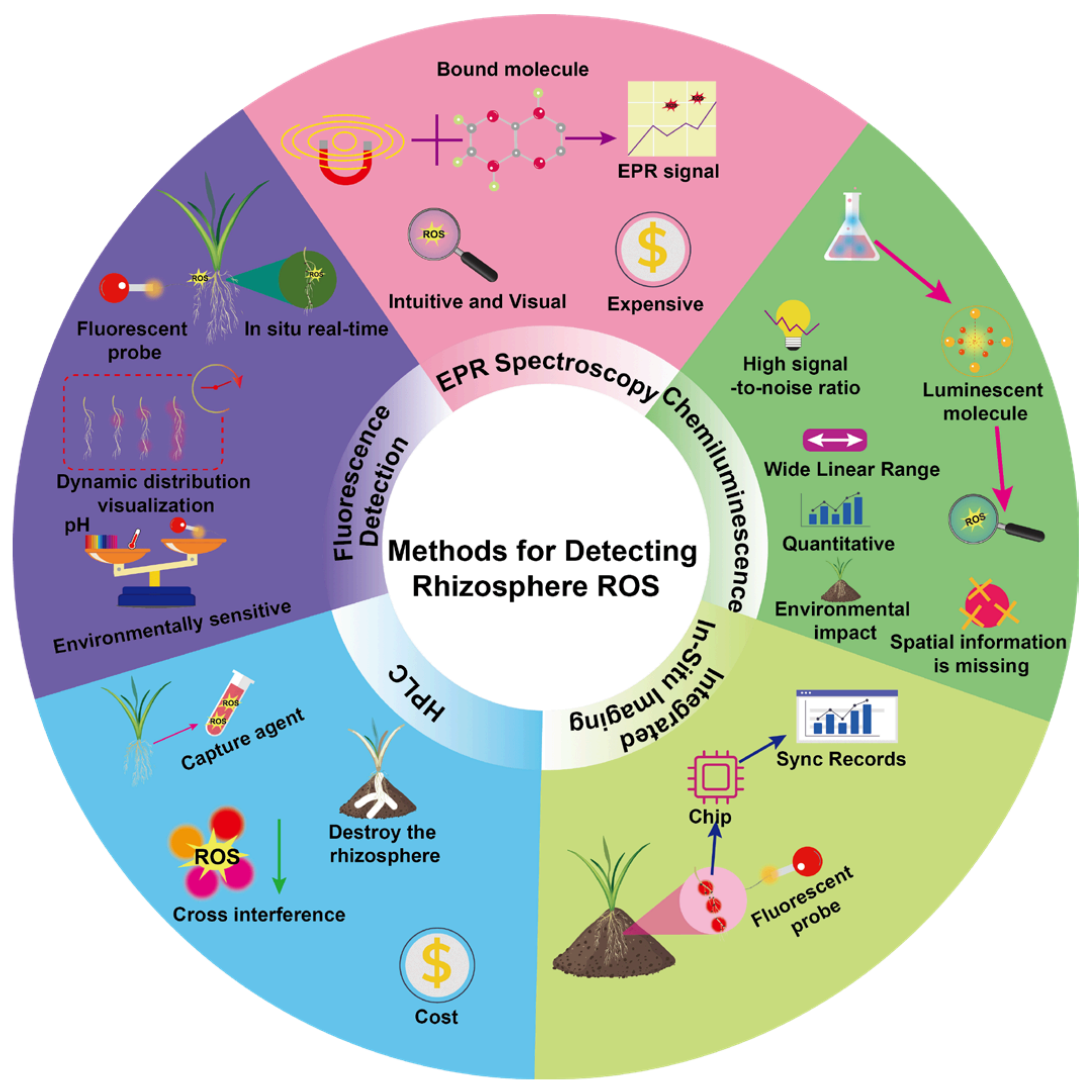
Detection methods and corresponding characteristics of rhizosphere ROS.

Overall, each rhizosphere ROS detection method possesses unique merits and drawbacks, with distinct applicable scenarios and analytical performance (Figure 1). In actual rhizosphere ROS studies, it is often difficult to meet the research needs by relying on only one detection technology. Different detection technologies need to be combined for complementary advantages. For example, fluorescence imaging detection technology can visually show the spatial distribution of rhizosphere ROS, and then the EPR method is used to verify the ROS types, realizing the comprehensive acquisition of multi-dimensional information such as the content, distribution, and existing forms of rhizosphere ROS [41,42]. Despite increasing studies on rhizosphere ROS, the field is marked by significant inconsistencies and knowledge gaps. For instance, reported ROS concentrations and distribution patterns often vary widely across studies [13,19,41]. These discrepancies are frequently attributed to the use of different detection methods. The choice of detection method fundamentally shapes the research outcome and is a primary source of controversy in the field. Fluorescence imaging provides invaluable spatial data but may suffer from quantification issues [16]. Although chemiluminescence provides sensitive quantification, it cannot capture spatial information [32]. EPR offers definitive radical identification yet lacks the ability to quantify ROS [37,38]. The lack of a standardized rhizosphere ROS detection means that comparing ROS contents across different studies is problematic. Future research should prioritize the development of multi-modal approaches that combine the strengths of these techniques to cross-validate findings.

## 3. Characteristics of Rhizosphere ROS

**Figure 2:**
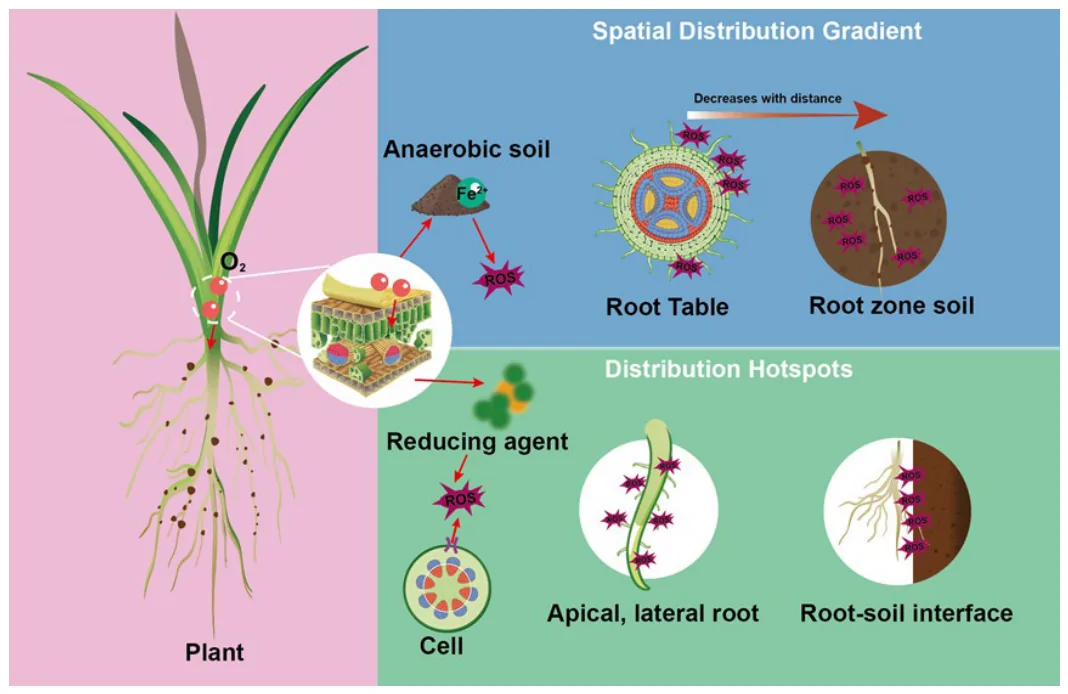
Spatial gradient and hotspot distribution of rhizosphere ROS.

Rhizosphere ROS exhibit heterogeneous spatial gradients and hotspot distribution patterns driven by coupled factors including root morphology, radial oxygen loss, root exudates, microbial communities, and soil minerals (Figure 2).

### 3.1. Spatial Distribution Gradient

As a typical micro-interface with prominent spatial heterogeneity, the rhizosphere displays orderly gradient changes in ROS distribution. Specifically, ROS are mainly concentrated in the root surface and the rhizosphere soil, and show a clear radial gradient pattern of decreasing concentration from the root surface to the surrounding soil [41]. This is closely related to the root structure of the plant, the radial oxygen loss process, and the diffusion and migration processes of rhizosphere ROS in the soil matrix [43]. Plants deliver oxygen from aerial tissues to root surfaces via radial oxygen loss. After that, root-released oxygen diffuses into surrounding anoxic soil and triggers sequential oxidation reactions mediated by soil reductants, including ferrous and manganous minerals and microorganisms, to boost ROS production [19,41]. Therefore, the content of ROS is the highest at the root surface. As the radial oxygen loss intensity gradually decreases with increasing distance from the root surface, and the rhizosphere soil matrix adsorbs and scavenges ROS, the ROS concentration gradually decreases, forming a significant radial gradient distribution pattern. Studies have shown that in the rice rhizosphere, the ROS concentration at the root surface is 3–5 times higher than that in the bulk soil 2 mm away from the root surface [19]. This radial gradient distribution pattern is not unique to the rice rhizosphere, but exists universally in the rhizospheres of various plants, including model plants such as Arabidopsis thaliana and crops such as maize. However, the radial gradient intensity and distribution range vary greatly among different plant species [44,45,46]. This difference is mainly related to the root structure, the radial oxygen loss intensity, and the composition of root exudates. For example, the roots of maize have a weaker ability to transport oxygen to the rhizosphere, and the radial oxygen loss intensity is significantly lower than that of rice [47]. Therefore, the radial gradient of ROS concentration in the maize rhizosphere is relatively gentle. For maize cultivated at 45% field water capacity, root surface ROS concentrations are roughly twice those of surrounding bulk soil, and the gradient change is concentrated in the range of 0–2 mm from the root surface [45]. However, the extent of this radial gradient is a subject of debate. While some studies report a steep decline within micrometers from the root surface [41], others using different techniques observe a more gradual decrease over several millimeters [44]. This controversy likely stems from differences in root hair density, mucilage production, and the microscale heterogeneity of soil pores, factors that are difficult to control and measure consistently.

### 3.2. Distribution Hotspots

Root tips and lateral roots constitute major rhizosphere ROS hotspots. This is because the root tip and lateral root are the most active areas for root physiological activities, with strong radial oxygen loss ability and abundant root exudates containing organic acids, phenols, and other reducing substances [46,48,49]. In addition, the cell wall structure in these areas is loose, and the respiratory burst oxidase homologs on the cell membrane are highly expressed, which can catalyze the generation of a large number of ROS [50,51]. Moreover, the microbial population in these two areas is dense, and the microbial extracellular respiration activity is strong, which will further promote the generation of ROS. In addition, ROS accumulate prominently at the root–rhizosphere soil interface [52,53]. Notably, rhizosphere ROS hotspots shift dynamically with plant developmental stages and diurnal cycles. The most obvious is the diurnal fluctuation of ROS in the rice rhizosphere [19]. The ROS concentration in the rice rhizosphere gradually increases from the morning, reaches its peak between 12:00 and 14:00 at noon [19], and then gradually decreases [54,55]. At night, the photosynthesis of the above-ground part stops and the root oxygen transport capacity decreases, resulting in a significantly lower ROS concentration at night than during the day [19,56,57]. The ROS hotspots are also not static. While root tips are generally accepted as hotspots, their activity fluctuates diurnally and in response to localized nutrient patches. A significant unresolved question is how these microscale, transient hotspots integrate to influence macroscale biogeochemical processes. Most current studies provide a snapshot in time, failing to capture the dynamic nature of these hotspots.

## 4. Coupled Production Mechanisms of Rhizosphere ROS

**Figure 3:**
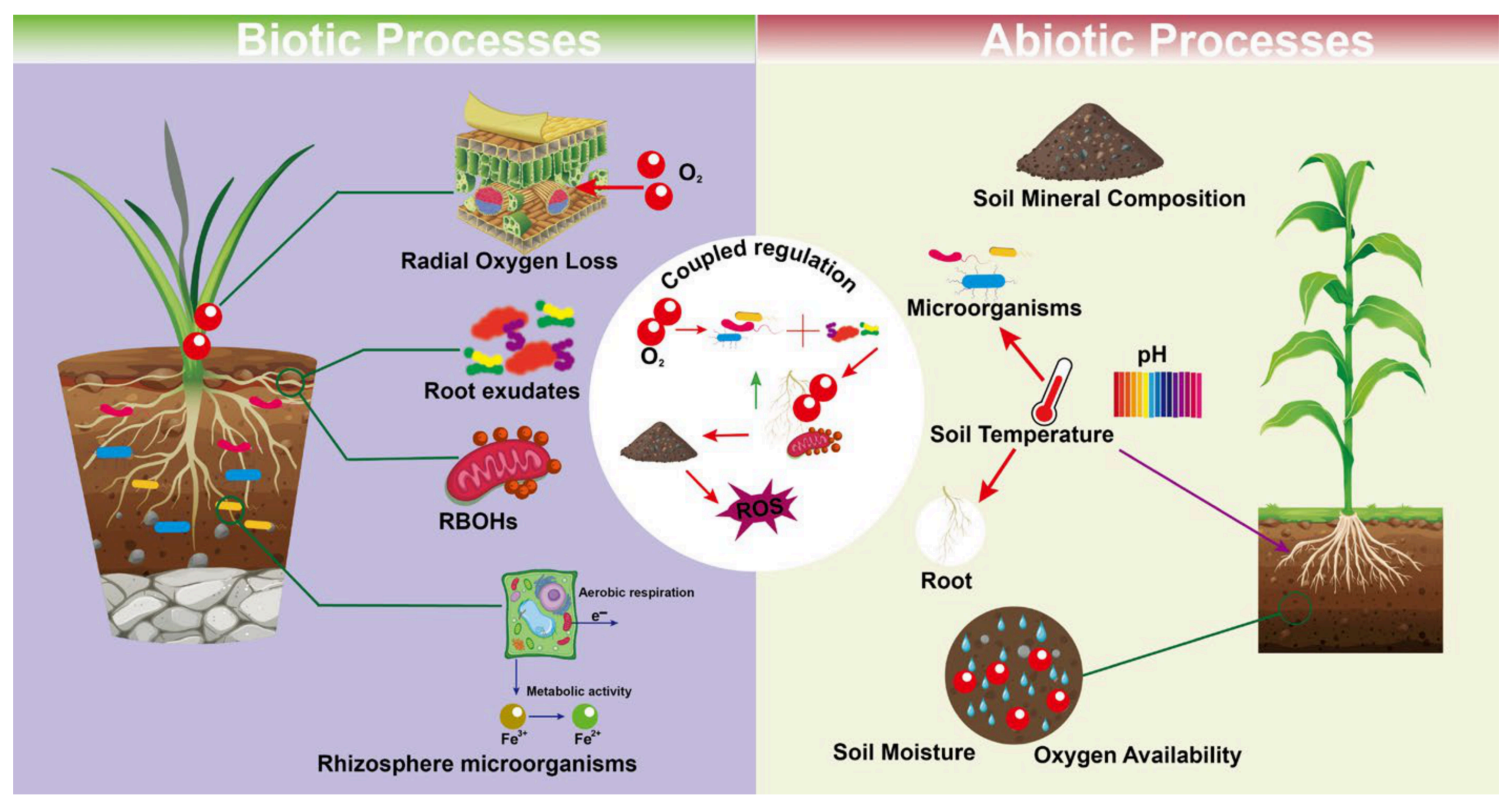
Coupled regulatory network of biotic and abiotic processes controlling rhizosphere ROS production.

The generation of rhizosphere ROS is determined by the coupled interaction of biotic and abiotic processes (Figure 3). These processes form a complex multi-factor coupled regulation network, which regulates the generation of ROS by affecting the root radial oxygen loss process, the root exudate secretion pattern, and the microbial community structure.

### 4.1. Biotic ROS Generation

The biological generation of rhizosphere ROS is jointly driven by root activity and microbial metabolism. The production of rhizosphere ROS is directly governed by root physiological activity, which is mainly achieved through radial oxygen loss and the secretion of root exudates. Radial oxygen loss from roots is the prerequisite for ROS generation in the rhizosphere. Through the radial oxygen loss process, oxygen is released into the surrounding rhizosphere soil and reacts with electron donors, promoting the generation of ROS [53]. The intensity of root radial oxygen loss is directly proportional to the generation rate of ROS. Studies conducted under flooded rice paddy conditions demonstrate that radial oxygen loss from rice roots accounts for over 30% of the total oxygen transported by root tissues, and ROS derived from this oxygen leakage contributes more than 40% of the total rhizosphere ROS pool [58]. In addition, root exudates play a critical role in regulating rhizosphere ROS formation. On the one hand, some enzymes in root exudates, such as peroxidases and oxidases, can directly catalyze the generation of ROS [59]. On the other hand, the organic acids, phenols, and reducing sugars in root exudates can act as electron donors and transfer electrons to oxygen, promoting the generation of ROS [60,61,62,63]. Meanwhile, root exudates can also provide a carbon source and energy source for rhizosphere microorganisms, regulating the growth, reproduction, and metabolic activity of microorganisms, and indirectly affecting the generation of ROS [64,65,66,67,68,69].

Microorganisms also exert crucial effects on the generation of rhizosphere ROS. Aerobic metabolism carried out by heterotrophic bacteria, iron-oxidizing microbes, and mycorrhizal fungi consumes oxygen within the rhizosphere matrix, which accelerates the outward diffusion of oxygen exuded from root tissues, indirectly promoting the generation of ROS [70]. In addition, the extracellular electron transfer process of microbial respiration will promote the conversion of oxygen molecules into ROS. Moreover, the metabolic activity of microorganisms can also regulate the concentration of electron donors such as Fe(II) in the rhizosphere soil, and regulate the generation of ROS by affecting the electron transfer process [71]. Notably, different rhizosphere microorganisms have significantly different effects on ROS generation. In the rhizosphere of rice, the iron-oxidizing bacteria in the rhizosphere soil can oxidize the Fe(II) in the soil to Fe(III), and the electrons released during the oxidation process can react with the oxygen to generate ROS [72,73]. In addition, some beneficial rhizosphere microorganisms, such as Bacillus, can also induce the plant root system to produce more ROS through interaction with root surface cells [74]. More importantly, the effect of microorganisms on the generation of rhizosphere ROS is not unidirectional. The metabolic activity of microorganisms can also regulate the dynamic balance of rhizosphere ROS by affecting the clearance process of ROS. For example, rhizosphere microorganisms can secrete antioxidant enzymes such as catalase and superoxide dismutase [75]. These enzymes can convert the excessively generated ROS into non-toxic substances such as oxygen and water, avoiding oxidative damage to the roots and the microorganisms themselves caused by excessively high ROS concentrations in the rhizosphere.

### 4.2. Abiotic ROS Generation

Abiotic ROS production is primarily driven by fluctuating soil physicochemical properties and mineral-catalyzed redox reactions [76]. Rhizosphere environmental perturbations, including drying–rewetting cycles, sharp fluctuations in redox potential (Eh), and pH imbalance, substantially disrupt the inherent physicochemical homeostasis of rhizosphere microenvironments [77,78,79]. Specifically, the re-oxygenation of waterlogged rhizosphere soil induces rapid oxygen reduction reactions, causing a burst of ROS within a short period [79]. Soil moisture exerts a dual constraint on rhizosphere ROS production. Under waterlogged conditions, soil pores become saturated, drastically reducing oxygen diffusion and suppressing ROS generation despite elevated anaerobic microbial activity. Conversely, severe drought impairs root physiological activity and limits exudate secretion, thereby inhibiting both direct enzymatic ROS production and indirect microbially mediated pathways. Consequently, peak ROS concentrations are observed at intermediate moisture levels, such as approximately 45% of field water capacity in maize, where oxygen supply and root metabolic activity are jointly optimized [13,76,77,78,79]. Moreover, temperature acts as an environmental factor governing rhizosphere ROS production, and moderate temperature (25 °C) maximizes ROS accumulation by facilitating the release of root exudates [13,80,81]. Furthermore, soil oxygen availability acts as a precursor to mediate ROS generation [13]. In addition to environmental regulation, catalytic redox reactions mediated by variable-valence metal minerals, predominantly iron and manganese oxides, represent the dominant pathway for abiotic ROS production in the rhizosphere, among which iron-driven Fenton and Fenton-like reactions make the most critical contribution [82,83]. Soil mineral composition fundamentally shapes both the capacity for ROS generation and the propagation of oxidative reactions. Redox-active minerals such as Fe- and Mn-oxides serve as electron donors and shuttles, while clay minerals with high surface area concentrate Fe(II) and organic reductants at reactive interfaces, thereby amplifying ROS production [83]. Specifically, plant roots continuously release reducing exudates, including soluble organic carbon, phenolic compounds, and low-molecular-weight organic acids, which provide abundant electrons for the rhizosphere micro-interface and reduce insoluble Fe(III) in soil iron minerals to soluble and reactive Fe(II) [84,85,86]. The generated ferrous ions subsequently initiate a series of chain redox reactions with ambient oxygen, producing ROS. Furthermore, these reducing root exudates can be directly and spontaneously oxidized by dissolved oxygen in the rhizosphere soil solution via non-enzymatic reactions, which acts as an auxiliary source of abiotic ROS [86]. In addition, these minerals can also act as electron shuttles to promote the electron transfer process between root exudates, microorganisms, and oxygen molecules, thereby promoting the generation of ROS. For example, in the rice rhizosphere, the Fe(II) in the soil can undergo a Fenton-like reaction with the oxygen released by the roots, generating ROS [86]. Moreover, some clay minerals can also promote the generation of ROS by adsorbing Fe(II) and other reducing substances on their surfaces, increasing the contact area between these substances and oxygen molecules [86].

### 4.3. Coupled Biotic and Abiotic ROS Generation

In fact, rhizosphere ROS production is not controlled by individual biotic or abiotic factors, but stems from their combined coupled interplay. These factors constitute a coupled regulatory network that regulates rhizosphere ROS production [87,88]. The main coupled regulation is the interactive system among root exudates, rhizosphere microbes, and soil mineral components [89]. Specifically, the oxygen released by the root system through the radial oxygen loss process and the organic acids and other reducing substances secreted in the form of root exudates are the basis for the generation of rhizosphere ROS. The rhizosphere microorganisms use the organic matter in the root exudates as a carbon source and energy source to carry out metabolic activities such as aerobic respiration. On the one hand, this process consumes oxygen in the rhizosphere environment, promoting the further diffusion of oxygen released by the root system to the surrounding soil. On the other hand, it secretes oxidases such as peroxidases, which catalyze the oxidation reaction of soil minerals and organic matter and promote the generation of ROS. The redox-active minerals in the soil, such as iron and manganese minerals, act as electron shuttles in this process [90]. They not only interact with the root exudates to promote the generation of ROS, but also affect the activity of rhizosphere microorganisms by regulating the concentration of oxygen and nutrients in the rhizosphere environment, further regulating the generation of ROS.

A major limitation of current research is the tendency to study biotic and abiotic processes in isolation. While the coupled regulation network is acknowledged, quantifying the relative contribution of each factor (e.g., root exudates, microbial activity, and soil minerals) under realistic field conditions remains a significant, unresolved challenge. Most models are based on simplified laboratory systems and may not accurately reflect the complexity of natural soils.

### 4.4. Molecular Regulatory Pathways of Rhizosphere ROS Production

Beyond macroscopic biotic and abiotic regulators, the generation of rhizosphere ROS is controlled by molecular cascades in plant roots and rhizosphere microorganisms. Plasma membrane-localized respiratory burst oxidase homologs (RBOHs) act as the main molecular regulators of root-derived ROS production [65,91]. In root cells, RBOH proteins catalyze the oxidation of cytosolic NADPH and transfer electrons to extracellular oxygen, generating extracellular O_2_^•−^ that is rapidly converted into H_2_O_2_ in the rhizosphere [51,92]. The transcription and post-translational modification of RBOHs are tightly modulated by upstream signaling events, including Ca^2+^ influx, mitogen-activated protein kinase cascades, and ROS-dependent auto-feedback loops [93]. Under normal growth conditions, basal RBOH activity sustains low levels of ROS to support root development. In response to rhizosphere environmental fluctuations, however, rapid RBOH activation triggers transient ROS bursts to initiate downstream biological responses. Microbe-derived rhizosphere ROS are also governed by specific molecular regulatory pathways. Beneficial rhizosphere bacteria and fungi encode conserved NADPH oxidases and extracellular oxidoreductases. Root exudates activate microbial pattern recognition receptors, which trigger intracellular signaling cascades and upregulate the expression of ROS-generating enzyme genes [92]. Meanwhile, microbial iron–sulfur cluster proteins mediate extracellular electron transfer, coupling soil mineral redox reactions with microbial metabolism to drive continuous ROS production at the rhizosphere micro-interface. These microbial regulatory pathways synergize with plant RBOH-dependent pathways, collectively shaping the spatiotemporal distribution patterns of rhizosphere ROS [93]. While the RBOH pathway is well-characterized in model plants, its regulation in diverse crop species under various environmental stresses is poorly understood. It is controversial whether the same molecular pathways dominate in all plant–microbe–soil systems. Mechanistic insights from Arabidopsis are often applied to wheat or maize without direct validation. There is a need for experimental studies that focus specifically on these crop plants.

To avoid irreversible oxidative damage caused by excessive ROS accumulation, plant roots and rhizosphere microbes evolve matched ROS-scavenging molecular networks that dynamically counterbalance ROS production. Enzymatic antioxidant systems constitute the dominant clearance machinery, including superoxide dismutase (SOD) that converts superoxide radical into hydrogen peroxide, catalase (CAT) and class III peroxidases that decompose hydrogen peroxide into water and molecular oxygen [13,41]. Non-enzymatic antioxidants such as ascorbate, glutathione, and phenolic root exudates also participate in neutralizing free radical species via electron donation [93]. The transcription of antioxidant enzyme genes is oppositely regulated by ROS signaling. The dynamic balance between ROS production and ROS scavenging by these antioxidant systems ultimately determines the spatiotemporal dynamics of ROS signaling in the rhizosphere, allowing it to function as a precise molecular switch rather than a source of indiscriminate damage.

Notably, rhizosphere ROS do not act as independent signal messengers but engage extensive molecular cross-talk with nitric oxide (NO) and phytohormones to coordinate root physiological and microbial symbiotic processes [92]. Low-concentration NO mediates S-nitrosylation modification to inhibit RBOH enzymatic activity and restrict excessive ROS bursts, whereas moderate NO synergizes with ROS to activate plant systemic defense against pathogenic microbes [92]. Multiple phytohormones also interconnect with ROS molecular cascades [93]. Abscisic acid triggers Ca^2+^ channel opening to boost RBOH-derived ROS under drought stress. Auxin relies on gradient ROS signals to guide root directional growth and lateral root primordia formation. Salicylic acid and jasmonic acid amplify ROS-mediated immune responses upon pathogen colonization. These signal molecules form intertwined regulatory loops with ROS, jointly determining whether rhizosphere ROS exert developmental signaling functions or toxic oxidative effects. While the RBOH-dependent ROS generation cascade has been thoroughly characterized in Arabidopsis thaliana model plants, large knowledge gaps remain regarding the conservation and differentiation of these molecular pathways in staple crop species under complex combined environmental stresses. Persistent controversy exists over whether identical ROS signaling and antioxidant regulatory networks dominate plant–microbe–mineral interactions across wetland rice, upland maize, and wheat. Most current mechanistic conclusions derived from Arabidopsis are frequently extrapolated to grain crops without direct genetic or biochemical validation under field rhizosphere conditions. Further targeted multi-omics and gene functional verification experiments focused on major agricultural crops are urgently required to resolve these unresolved molecular regulatory discrepancies.

The maintenance of ROS homeostasis on the root surface is a highly complex and context-dependent process, and the mechanisms governing this balance remain incompletely understood. This complexity arises from several intertwined layers. First, the net ROS level at the root surface is determined not by a single pathway but by the simultaneous and often competing activities of multiple generation systems (e.g., RBOHs, secreted peroxidases, and abiotic Fenton chemistry) and scavenging systems (e.g., SOD and CAT) [61,66]. The relative contribution of each component shifts dynamically with developmental stage, tissue type, and environmental conditions, making it difficult to define a universal regulatory mechanism. Second, the induction signal, including biotic (e.g., pathogen-associated molecular patterns, symbiotic signals), abiotic (e.g., drought, nutrient deficiency, and metal toxicity), and developmental (e.g., lateral root initiation), triggers entirely distinct signaling cascades that converge on ROS metabolism through different regulatory nodes [13]. For instance, immune responses rapidly activate RBOHs via Ca^2+^ influx and phosphorylation, whereas drought-induced ROS bursts are primarily mediated by abscisic acid signaling [63,67]. These parallel pathways often engage different scavenging components and exhibit distinct spatiotemporal dynamics, leaving a systematic framework to predict how a given signal is translated into a specific ROS signature still to be developed. Third, the tight coupling between plant roots and the rhizosphere microbiome further complicates this regulation. Microbial-derived signals, quorum-sensing molecules, and even microbial antioxidant secretions can directly modulate the plant’s ROS machinery, and the outcome depends on the intensity, duration, and spatial pattern of the ROS response [73,74]. To date, our understanding of how these biotic and abiotic signals are integrated at the molecular level to achieve precise ROS balance remains largely descriptive. Addressing this gap will require the development of real-time, high-resolution ROS imaging techniques compatible with complex soil environments, combined with genetic tools that allow conditional manipulation of individual generation and scavenging components in specific root cell types.

## 5. Environmental Effects of Rhizosphere ROS

**Figure 4:**
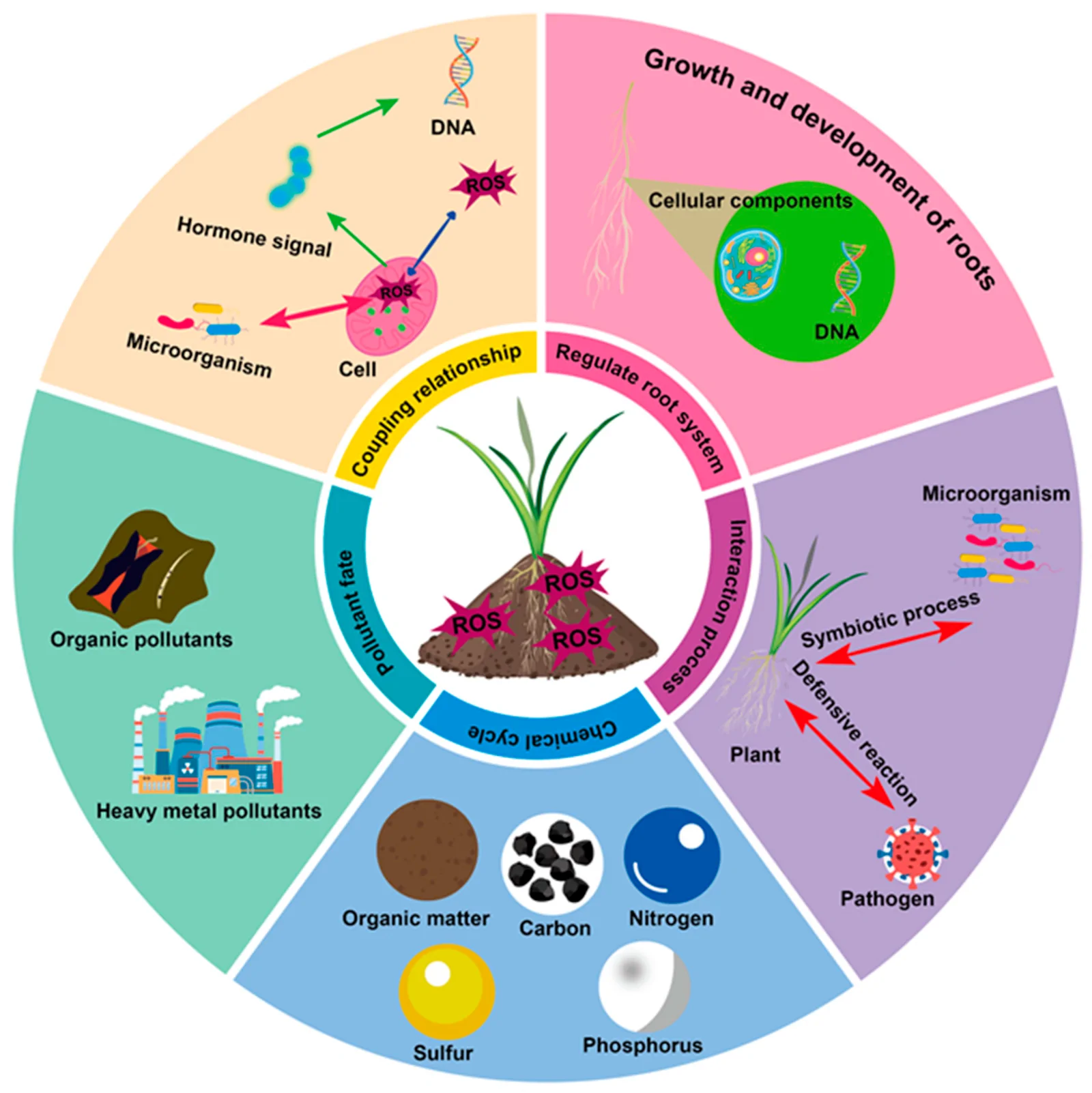
The environmental effects induced by rhizosphere ROS.

Rhizosphere ROS exert diverse environmental impacts (Figure 4). Serving as vital signaling molecules, ROS modulate root growth and development, facilitate the establishment of plant–microbe symbiosis, and enhance plant tolerance against external stressors. Meanwhile, as a strong oxidizing agent, ROS drive the biogeochemical cycles of elements in the rhizosphere soil and mediate the transformation and degradation of organic contaminants and heavy metals.

### 5.1. Regulation of Root Growth and Development

As a key signaling substance, ROS can regulate the growth and development of plant roots, the formation of root hairs, and the root tip growth process. The regulation of root growth and development by rhizosphere ROS is mainly achieved by regulating the expression of root-related genes and the dynamic balance of root cell wall components [91,94]. Meanwhile, ROS can regulate the growth and development of the root system and the formation of root hairs. Specifically, the ROS generated in the root tip and lateral root can act as a signaling molecule to regulate the proliferation and differentiation of root cells, promote the formation of lateral roots and root hairs, and increase the root surface area. This helps plants absorb more water and nutrients from the soil. In addition, the ROS generated in the root hair growth zone can regulate the tip growth process of root hairs by regulating the activity of Ca^2+^ channels on the cell membrane [95]. The gradient distribution of rhizosphere ROS can also affect the root system’s perception of the soil’s water and nutrient status, thereby regulating the root system’s growth orientation and nutrient absorption efficiency.

### 5.2. Regulation of Plant–Microbe Interactions

ROS play a key role in the process of plant–microbe interactions, which mainly include modulating the host plant’s defense response against pathogenic microorganisms, regulating the symbiosis process between plants and beneficial microorganisms, and structuring the construction of the rhizosphere microbial community structure. As a key component of plant innate immunity, rhizosphere ROS orchestrate root defense against invasive pathogens. When pathogenic microorganisms invade the root system from the rhizosphere soil, the pattern recognition receptors on the root cell membrane can specifically recognize the pathogen-associated molecular patterns on the surface of the pathogenic microorganisms [96,97]. Through the Ca^2+^ signaling pathway and the RBOHs enzyme pathway, the root system will trigger a rapid and intense ROS burst [66,98]. A large amount of ROS will be released to the rhizosphere, killing the pathogenic microorganisms or inhibiting their growth [65]. Meanwhile, the ROS burst will also act as a signal to stimulate the root system to synthesize more defense-related enzymes and secondary metabolites, activating the plant’s systemic defense response to prevent the further spread of pathogenic microorganisms. Moreover, rhizosphere ROS also play an important regulatory role in the establishment of symbiotic relationships between plants and beneficial microorganisms. The rhizosphere is home to a large number of beneficial microorganisms, such as mycorrhizal fungi and plant growth-promoting rhizobacteria [99,100]. These beneficial microorganisms can promote plant growth and enhance the plant’s resistance to stresses such as drought and salinity. In the early stage of symbiosis establishment between plants and these beneficial microorganisms, ROS will be generated in the rhizosphere. It will act as a signaling molecule to participate in the symbiosis establishment process and promote the recognition and colonization of beneficial microorganisms by the root system. More importantly, rhizosphere ROS can regulate the construction of the rhizosphere’s microbial community structure. Different types of rhizosphere microorganisms have significantly different tolerances to ROS. For example, beneficial microorganisms such as Bacillus and mycorrhizal fungi have strong antioxidant enzyme activity and can maintain metabolic activity in a high-ROS environment [101]. However, some pathogenic microorganisms and anaerobic microorganisms are very sensitive to ROS [102]. Therefore, the ROS concentration gradient in the rhizosphere will form a unique selective pressure, which will selectively promote the growth and reproduction of beneficial microorganisms and inhibit the growth and reproduction of pathogenic microorganisms and anaerobic microorganisms. This will shape the rhizosphere microbial community structure that is beneficial to plant growth.

Notably, the outcome of ROS-mediated plant–microbe signaling is strongly modulated by microbiome composition and the compensatory dynamics within the community [100]. Taxa with high antioxidant secretion capacity can dampen localized ROS bursts and facilitate benign colonization, whereas the loss of such taxa may unmask stronger oxidative responses [99]. Furthermore, functional redundancy within microbial consortia means that inhibition of a single ROS-generating pathway may be compensated by alternative microbial routes, complicating efforts to establish causal links between specific microbial members and net rhizosphere ROS levels [101,102].

### 5.3. Driving Biogeochemical Cycles

ROS are the most important active oxidizing agents, playing a key role in the biogeochemical cycles of carbon, nitrogen, and phosphorus [103]. Specifically, the oxidative properties of ROS can directly drive the decomposition and mineralization of soil organic matter and promote the release of carbon, nitrogen, phosphorus, and other nutrient elements from organic matter. In addition, rhizosphere ROS can also indirectly regulate the biogeochemical cycle of elements by affecting the activity of microorganisms and the redox process of soil minerals. For example, the oxidation of Fe(II) mediated by rhizosphere ROS can promote the conversion of iron and manganese minerals in the soil, releasing the adsorbed phosphorus and improving the effectiveness of phosphorus supply [103].

### 5.4. Promoting the Transformation and Degradation of Pollutants

Rhizosphere ROS accelerate the transformation and degradation of diverse organic and heavy metal contaminants. They are one of the most important abiotic degradation-driving forces in the rhizosphere environment. This effect provides an important basis for the phytoremediation of contaminated soil [82,104,105]. For example, in the rhizosphere of plants, ROS can directly oxidize and degrade organic pollutants such as polycyclic aromatic hydrocarbons, petroleum hydrocarbons, and pesticides remaining in the soil, converting them into low-toxic or non-toxic small-molecule substances such as carbon dioxide and water [45,53]. In addition, ROS can also regulate the form transformation of heavy metal pollutants in the soil, reducing their bioavailability and toxicity. For example, in the rhizosphere of rice, ROS can oxidize As(III) to As(V), reducing the toxicity and migration ability of arsenic [106,107]. However, it is worth noting that the environmental effects of rhizosphere ROS are not always positive. Under certain conditions, ROS may also have negative impacts on the rhizosphere and the ecological environment. For example, excessively high concentrations of ROS may cause oxidative damage to the root system, destroying the normal structure and function of the root system [108,109]. In addition, excessively high concentrations of ROS may also have a negative impact on the community structure of beneficial microorganisms in the rhizosphere, reducing the abundance of beneficial microorganisms and weakening the rhizosphere’s ecological defense function. Therefore, in the process of applying rhizosphere ROS to the remediation of contaminated soil, it is necessary to comprehensively consider the dual effects of ROS and take appropriate regulatory measures to avoid the negative impacts of ROS on the rhizosphere.

### 5.5. The Coupling Relationship Between Rhizosphere ROS and Downstream Signal Networks

Rhizosphere ROS serve as pivotal secondary messengers that couple intracellular and interspecific signaling networks to modulate diverse biological processes. Rhizosphere ROS can penetrate root cell walls and plasma membranes, thereby activating intracellular ROS signaling cascades. Specifically, ROS-induced oxidative modification triggers downstream activation of mitogen-activated protein kinase 3/6 and calcium-dependent protein kinase modules [110,111]. These signaling modules further regulate the transcription of genes associated with root growth, stress tolerance, and hormone biosynthesis, including auxin, abscisic acid, and salicylic acid. The integrated ROS–hormone signaling cascade forms a closed regulatory loop that coordinates root morphogenesis and rhizosphere environmental adaptation.

Furthermore, rhizosphere ROS mediate bidirectional signal communication between plants and their associated rhizosphere microbiota. Low concentrations of ROS act as symbiotic signals to induce microbial colonization-related genes, facilitating the establishment of beneficial plant–microbe symbiosis. In contrast, excessive ROS accumulation initiates oxidative stress signaling, upregulating plant defense genes and microbial stress-responsive pathways [112,113]. This process suppresses the proliferation of pathogenic microbes and remodels the assembly of rhizosphere microbial communities. Such concentration-dependent dual regulatory mechanisms enable rhizosphere ROS to balance plant growth promotion and stress resistance, serving as a core molecular hub that connects rhizosphere physiochemistry, microbial metabolism, and plant physiological responses. Therefore, a dynamic balance between ROS generation and scavenging is essential for maintaining normal ROS signaling functions. Plant roots and rhizosphere microorganisms possess sophisticated antioxidant systems, including superoxide dismutase, catalase, and peroxidase [114,115]. These antioxidant enzymes eliminate excess ROS to prevent irreversible oxidative damage, while retaining basal ROS levels to ensure effective signal transduction. The precise coordination between ROS-producing molecular pathways and antioxidant regulatory networks ultimately determines the biological and ecological functions of rhizosphere ROS.

Notably, existing research on rhizosphere ROS environmental effects suffers from prominent limitations. First, most experiments adopt high-concentration exogenous ROS treatments that deviate from natural microscale ROS gradients in real rhizospheres, leading to artificially amplified phenotypes inconsistent with in situ conditions. Second, studies commonly isolate plant, microbial, or mineral systems separately and fail to disentangle synergistic or antagonistic ROS effects within the intact plant–microbe–soil continuum, resulting in one-sided conclusions. These unresolved discrepancies hinder our capacity to predict rhizosphere ROS-mediated biogeochemical processes under variable agricultural and environmental scenarios.

## 6. Conclusions and Perspectives

This review systematically summarizes recent advances in rhizosphere ROS, focusing on detection techniques for rhizosphere ROS, spatial distribution properties, coupled regulatory mechanisms of biotic and abiotic factors, and environmental effects mediated by ROS. Based on the current knowledge and latest progress in the field, we propose the following research needs to provide an in-depth understanding of the production and impact of rhizosphere ROS.

### 6.1. Optimization of ROS Detection Techniques

Novel in situ detection techniques and integrated platforms should be developed to improve the spatial resolution, precision, and visualization of rhizosphere ROS measurement. The accurate quantification and precise localization of rhizosphere ROS are the prerequisites for deeply revealing the generation process and mechanism of ROS. On the one hand, existing detection approaches, including fluorescence imaging, require further optimization. By developing highly specific ROS-responsive probes and optimizing the optical path and signal acquisition system, the anti-interference ability and detection accuracy of the detection technologies in rhizosphere environments can be improved. On the other hand, a new integrated in situ platform for rhizosphere ROS monitoring should be developed by combining microfluidic chips and fluorescence imaging. This platform can achieve the synchronous acquisition of multi-dimensional information such as the content, distribution, and existing forms of rhizosphere ROS, realizing the real-time dynamic monitoring of rhizosphere ROS.

### 6.2. Mechanism of Rhizosphere ROS Production

Further research should focus on coupled regulatory mechanisms governing rhizosphere ROS production within plant–microbe–soil interactions. The generation of rhizosphere ROS is the result of the coupled interaction of multiple factors. Only by clarifying the coupled regulation mode and synergistic mechanism of the plant–microbe–soil interactions can we systematically reveal the essence of the rhizosphere ROS generation process. Therefore, it is necessary to use microelectrodes, high-throughput sequencing, and mass spectrometry to analyze the synergistic effects of plant–microbe–soil interactions on ROS production. Moreover, multi-factor coupling models of rhizosphere ROS formation that integrate multiple stresses, such as temperature, moisture, and pollutants, should be built to quantify the respective contributions, which helps systematically clarify the dominant drivers controlling rhizosphere ROS dynamics under complex field conditions.

### 6.3. Plant-Dependent ROS Regulation

More investigations should explore rhizosphere ROS processes and their regulatory mechanisms across diverse plant species. The existing research on rhizosphere ROS mainly focuses on model plants such as rice, and there is a lack of research on the rhizosphere ROS process of other plants, especially dryland crops and cash crops. Future research needs to focus on different plant species and systematically analyze the differences in the rhizosphere ROS generation process, distribution characteristics, and regulation mechanisms of different plants. Furthermore, the regulatory pathways through which rhizosphere ROS modulate plant growth and stress tolerance should be elucidated, laying a theoretical foundation for establishing rhizosphere management technologies targeted at efficient nutrient use and sustainable agricultural production.

### 6.4. Application of Rhizosphere ROS

Further research should be carried out on rhizosphere ROS regulation and its applications in environmental remediation. Rhizosphere ROS have important application potential in the fields of soil improvement, agricultural production, and the remediation of contaminated soil. On the one hand, the development of rhizosphere ROS regulation technologies needs more attention. For example, by screening and preparing beneficial rhizosphere microbial inoculants that can regulate the ROS level in the rhizosphere, and optimizing farmland management measures such as irrigation and fertilization, the generation of rhizosphere ROS can be regulated. This can promote the positive effects of ROS on the rhizosphere environment.

On the other hand, deeper exploration is required to investigate how rhizosphere ROS accelerate soil pollutant degradation and boost plant stress tolerance. The detection methodologies offer practical tools for monitoring ROS-based remediation. In situ microelectrodes enable real-time tracking of ROS concentrations, while EPR spectroscopy can confirm the generation of target radical species responsible for pollutant oxidation. Integrating these analytical approaches with remediation trials will be essential for moving beyond empirical application toward mechanism-based optimization. Relevant findings can provide technical support for creating efficient, safe, and eco-friendly rhizosphere regulation strategies. Moreover, the practical efficacy of ROS-mediated soil remediation is tightly coupled to edaphic properties. In clay-rich soils, high specific surface area concentrates Fe(II) and organic reductants, potentially amplifying ROS generation. However, the same clay surfaces may scavenge ^•^OH, shortening its effective diffusion radius and limiting pollutant accessibility. Soil organic matter exerts dual effects, supplying electrons to sustain Fenton cycling while also competing for oxidants. Furthermore, the strong pH dependence of Fenton chemistry (optimum pH 5.0–6.5) means that remediation efficiency in neutral or alkaline soils may require acidification pretreatment. These edaphic constraints highlight the need for site-specific optimization rather than universal application protocols. These advances will facilitate efficient rhizosphere resource utilization and advance sustainable agricultural development. Rhizosphere ROS represent only one component of the complex biogeochemical processes that contribute to soil remediation. Effective remediation strategies must integrate ROS-based approaches with other established methods, including phytostabilization, microbial biodegradation, and soil amendment applications. The optimal strategy will depend on site-specific edaphic characteristics, contaminant types, and remediation goals. Future research should aim to develop integrated models that predict remediation outcomes based on the coupled dynamics of soil properties, microbial communities, and ROS generation.

## Data Availability

No new data were created or analyzed in this study. Data sharing is not applicable to this article.
